# Local News

**Published:** 1885-05

**Authors:** 


### LOCAL NEWS.

CHICAGO MEDICAL COLLEGE.—The twenty-sixth annual commencement exercises of the Chicago Medical College, the Medical Department of the Northwestern University, were held in the Grand Opera House, on Tuesday, March 24th.

The degree of Doctor of Medicine was conferred by President Cummings of the University upon forty-one members of the graduating class, who were reported by the Dean of the College, Professor N. S. Davis, as having complied with all the prescribed requirements of the college to entitle them to that degree. The Secretary of the College, Professor Walter Hay, announced the names of the successful competitors for the different prizes offered. A banquet was tendered by the faculty of the college to the Alumni on Tuesday evening, at the Leland Hotel, at which Professor H. A. Johnson presided.

The regular course of lectures, for practitioners, began on the following day and continued four weeks.

